# Auditory driven gamma synchrony is associated with cortical thickness in widespread cortical areas

**DOI:** 10.1016/j.neuroimage.2022.119175

**Published:** 2022-07-15

**Authors:** Anna-Lisa Schuler, Giulio Ferrazzi, Nigel Colenbier, Giorgio Arcara, Francesco Piccione, Florinda Ferreri, Daniele Marinazzo, Giovanni Pellegrino

**Affiliations:** a IRCCS San Camillo Hospital, Via Alberoni 70, Venice 30126, Italy; b Department of Data Analysis, Faculty of Psychology and Educational Sciences, Ghent University; c Rehabilitation, University of Padua, Italy; d Unit of Neurology, Unit of Clinical Neurophysiology, Study Center of Neurodegeneration (CESNE), Department of Neuroscience, University of Padua, Padua, Italy; e Department of Clinical Neurophysiology, Kuopio University Hospital, University of Eastern Finland, Kuopio, Finland

**Keywords:** Magnetoencephalography (MEG), Synchrony, Auditory Steady State Responses (ASSR), Gamma, Cortical thickness, Cerebral cortex

## Abstract

**Objective:**

Gamma synchrony is a fundamental functional property of the cerebral cortex, impaired in multiple neuropsychiatric conditions (i.e. schizophrenia, Alzheimer's disease, stroke etc.). Auditory stimulation in the gamma range allows to drive gamma synchrony of the entire cortical mantle and to estimate the efficiency of the mechanisms sustaining it. As gamma synchrony depends strongly on the interplay between parvalbumin-positive interneurons and pyramidal neurons, we hypothesize an association between cortical thickness and gamma synchrony. To test this hypothesis, we employed a combined magnetoencephalography (MEG) – Magnetic Resonance Imaging (MRI) study.

**Methods:**

Cortical thickness was estimated from anatomical MRI scans. MEG measurements related to exposure of 40 Hz amplitude modulated tones were projected onto the cortical surface. Two measures of cortical synchrony were considered: (a) inter-trial phase consistency at 40 Hz, providing a vertex-wise estimation of gamma synchronization, and (b) phase-locking values between primary auditory cortices and whole cortical mantle, providing a measure of long-range cortical synchrony. A correlation between cortical thickness and synchronization measures was then calculated for 72 MRI-MEG scans.

**Results:**

Both inter-trial phase consistency and phase locking values showed a significant positive correlation with cortical thickness. For inter-trial phase consistency, clusters of strong associations were found in the temporal and frontal lobes, especially in the bilateral auditory and pre-motor cortices. Higher phase-locking values corresponded to higher cortical thickness in the frontal, temporal, occipital and parietal lobes.

**Discussion and conclusions:**

In healthy subjects, a thicker cortex corresponds to higher gamma synchrony and connectivity in the primary auditory cortex and beyond, likely reflecting underlying cell density involved in gamma circuitries. This result hints towards an involvement of gamma synchrony together with underlying brain structure in brain areas for higher order cognitive functions. This study contributes to the understanding of inherent cortical functional and structural brain properties, which might in turn constitute the basis for the definition of useful biomarkers in patients showing aberrant gamma synchronization.

## Highlights

• Significant correlation between auditory-driven gamma synchrony and cortical thickness
• Correlation prevalent in A1, but also in parts of the frontal cortex
• A1 auditory-driven gamma connectivity significantly associated with cortical thickness
• Correlation prevalent in the temporal, frontal, parietal and occipital lobes

## 1 Introduction

Gamma synchrony (GS) is the ability of the cerebral cortex to generate gamma activity in phase (Bartos et al., 2007; Traub et al., 2003, 1996; Whittington et al., 1995). GS can be driven by exposure to external stimuli, such as sounds modulated at 40 Hz: the phase of cortical oscillations becomes progressively aligned to the sound, resulting in an increased gamma synchronization (Adaikkan and Tsai, 2020). The phenomenon of neurons synchronizing with auditory cues was discovered in the eighties of the last century and termed Auditory Steady-State Response (ASSR). It was initially presumed that auditory-driven gamma synchronization was a phenomenon limited to the temporal/auditory cortex (Gutschalk et al., 1999; Pantev et al., 1996; Roß et al., 2000). Yet, recent evidence suggests that auditory-driven gamma synchronization rather involves the entire cortical mantle. This is supported by whole head EEG and MEG mapping (Farahani et al., 2021; Pellegrino et al., 2019b), invasive recordings in animal studies (Cho et al., 2015, 2020) and electrocorticography in epilepsy patients (Tada et al., 2021). As such, exposure to gamma sounds provides a viable way to investigate gamma synchronization of the entire cortical surface.

Abnormal gamma synchrony has been found in a wide range of neuropsychiatric conditions, i.e. autism spectrum disorder, schizophrenia, Alzheimer's disease, attention deficit/hyperactivity disorder, epilepsy and stroke (Başar, 2013; Başar et al., 2016; Herrmann and Demiralp, 2005; Kwon et al., 1999; Parciauskaite et al., 2021; Pellegrino et al., 2019a; Pellegrino et al., 2012; Uhlhaas and Singer, 2006). It is supposed that this is due to the fact that gamma synchrony reflects a fundamental property of information integration (Fries, 2009; Galarreta and Hestrin, 2002; Herrmann et al., 2010) and relies on the precise tuning of specific, complex and fragile circuits involving cortical and sub-cortical structures (Plourde et al., 2008). GS reflects glutamate/GABA balance and depends on the interaction between parvalbumin-positive (PV+) inhibitory interneurons (mostly layer 4 and 5) and pyramidal cells (mostly layer 3 and 5) (Adaikkan and Tsai, 2020; Gonzalez-Burgos and Lewis, 2012; Parker and Sweet, 2018). In detail, this circuit comprises: a) interconnected pyramidal cells, b) GAP-junctions, and c) GABAergic inhibitory interneurons able to fire at the pace of gamma band (Brenner et al., 2009; Javitt and Sweet, 2015; von Ellenrieder et al., 2016). The inhibitory neurons (PV+, fast-spiking GABA interneurons known as chandelier and basket cells) seem to play the most important role in this circuit. Animal models revealed that auditory-driven gamma synchrony involves all cortical layers, with maximum synchrony in the granular layer (layer 4 or thalamo-recipient layers) (Li et al., 2021). Subcortical regions are part of a large thalamo-cortical reverberant circuit and the thalamus acts as a pulse generator with its projections to the granular cortex (layer 4) (Chaitanya et al., 2020). Thalamic projections are mostly excitatory, but whether the net effect of thalamic activity is an enhancement of cortical synchrony is still unclear. Moreover, astrocytes and microglia were hypothesized to be involved in gamma entrainment (Adaikkan and Tsai, 2020; Shin et al., 2018; Williams et al., 2013).

The anatomical integrity and efficiency of the thalamo-cortical and PV+-pyramidal circuit, together with microglia and astrocytes, might be reflected in cortical thickness and thalamic volumes (Proskovec et al., 2020; Shin et al., 2018; Williams et al., 2013). Indeed, a correlation between pyramidal cell layer and cortical thickness was established in a post-mortem study (Williams et al., 2013), and further supported by a significant correlation between gene expression factors from the Allan Brain Atlas and cortical thickness in a later study (Shin et al., 2018).

In terms of structure-function relationship, a positive correlation between auditory-driven gamma synchrony and cortical thickness has been shown in the superior temporal gyrus in healthy subjects (Edgar et al., 2014; Kim et al., 2019) and in schizophrenic patients (Hirano et al., 2020).

Based on these observations, we hypothesize an association between brain structure and gamma synchrony. For this purpose, we performed an auditory-driven gamma synchronization paradigm during MEG and considered two measures of cortical synchrony: (a) inter-trial phase consistency (*ITPC*) at 40 Hz, providing a vertex-wise estimation of gamma synchronization, and (b) phase-locking values (*PLV*) between primary auditory cortices (A1) and the remaining cortical mantle, providing a measure of long-range cortical synchrony. Our main focus with respect to anatomical features was on cortical thickness and volume of thalamic nuclei, as these two features might be the most relevant for the generation of gamma synchronization.

## 2 Materials and methods

### 2.1 Participants and experimental design

**Fig. 1:**
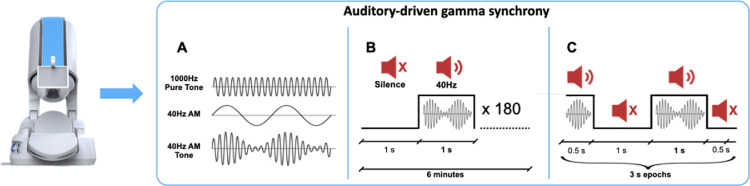
MEG Paradigm. Auditory stimulus. (A) An amplitude modulated tone was generated with the following parameters: Carrier Frequency = 1000 Hz, Amplitude Modulating Frequency = 40 Hz. (B) The paradigm employed to induce 40 Hz synchrony consisted of 180 repetitions lasting two seconds each. Each trial included 1 s of Silence followed by a 40 Hz amplitude-modulated (AM) tone, so that the stimulus onset interval was two seconds. (C) For ASSR analyses data were epoched in segments of 3 s (1.5 s before stimulus onset, 1 s of stimulus and 0.5 s after stimulus end).

Seventy-two scans from 52 healthy subjects (40 female, mean age=29.25 ± 7.43; min/max=21/57) were included in this study. We excluded subjects with: (b) hearing deficits; (a) contraindication to MRI; (c) taking medications acting on the central nervous system, (d) present or past neuropsychiatric disorders, and e) history of head trauma (Huang et al., 2020). The study followed the recommendations of the 1964 Declaration of Helsinki and was approved by the local Ethics Board (Venice Province). All participants signed a written informed consent prior to participation. The study design is illustrated in Fig. 1, 2. Briefly, all participants underwent two sessions: (1) an MEG recording during binaural exposure to 40 Hz amplitude modulated (AM) auditory tones; (2) T1-weighted anatomical MRI for measuring cortical thickness, thalamic volumes, and for building the head-specific model for MEG source imaging.

**Fig. 2:**
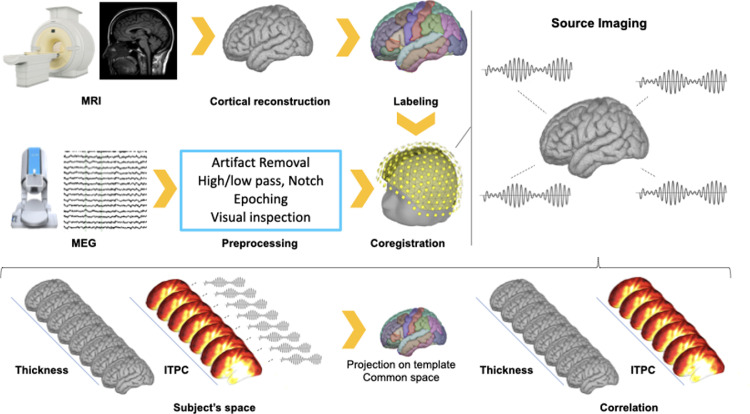
Analysis Pipeline. MEG recordings were performed with 275 gradiometers, and subsequently data was preprocessed including artifact removal, high- and low-pass filtering, epoching and visual inspection. MEG-data was co-registered on the cortical reconstruction of the MRI. Cortical thickness maps were extracted from the T1 weighted image and z-scores for *ITCP* at 40 Hz were extracted in source space. Both maps were then projected from subject space into MNI space and vertex-wise correlations were calculated between the two maps.

### 2.2 MEG session

MEG was performed in a shielded room with a CTF-system (MISL, Vancouver, Canada, 275 axial gradiometers). Eye movements and EKG were detected with dedicated bipolar electrodes. Data was sampled at 1200 Hz. Head movements during MEG scan were detected with a continuous head localization system capable of tracking the position of the coils placed on three anatomical landmarks (left and right preauricolar points and nasion) with high temporal resolution (Pellegrino et al., 2016). All subjects were asked to maintain a regular sleep cycle for at least three days prior to the MEG scan. MEG scans were performed with the participants lying down in supine position, with eyes closed. Participants were asked to relax and to not pay attention to the auditory sounds. 40 Hz sounds were given binaurally, with the CTF system, via MEG compatible plastic tubes connected to in-ear plugs. The sound intensity, tested with a Sound Level Meter at each in-ear headphone for each MEG recording, was 85 dB, in agreement with standard procedures (Kim et al., 2019; Larsen et al., 2018; Legget et al., 2017). All subjects heard the sound properly, balanced between left and right, and none of them reported significant discomfort. The 40 Hz amplitude-modulated tone was designed on a carrier frequency of 1000 Hz, and set to have a 6 ms fade-in and fade-out period; clipping was prevented through normalization (Ross et al., 2003; Roß et al., 2000). The 40 Hz sound was generated in MATLAB (The Mathworks, v. 2016b) according to the formula:

$$A=\sin\left(2\pi f_{c}t\right)*\left(1+m*\cos\left(2\pi f_{m}t\right)\right)$$

where *A* is the amplitude, *f_c_* is the carrier frequency set to 1000 Hz, *m* is the modulation depth, *f_m_* is the frequency of modulation, set to 40 Hz and *t* is the vector of time points for one second of stimulus, at a sampling rate of 44100 Hz. The duration of the sound was 1 s, interleaved with 1 s of Silence, so that the duration of each trial was 2 s (Fig. 1). 180 trials were delivered, for an overall 6 min duration. The entire sequence was coded and delivered with the PsychoPy toolbox (http://www.psychopy.org/) (Peirce, 2009, 2007). For gamma synchrony studies the precision of the trigger system is of utmost importance, therefore we dedicated an additional MEG analogical channel to the recording of the sound actually delivered to the subject. This signal could then be used to adjust and realign the digital triggering.

### 2.3 MRI session

The brain MRI was performed with a 1.5 T Achieva Philips scanner (Philips Medical Systems, Best, The Netherlands). A 3-dimensional Magnetization Prepared Rapid Gradient Echo (MP-RAGE) T1-weighted scan was acquired using an 8-channel receiver head coil with the following parameters: repetition time [TR]=8.3 ms, echo time [TE]=4.1 ms, flip angle=8°, isotropic spatial resolution=0.87 mm.

### 2.4 Analysis pipeline

#### 2.4.1 MRI analysis

Segmentation/reconstruction and labeling, as well as estimation of cortical thickness of the anatomical MRI, were performed with the CAT12 toolbox for MATLAB implemented in the Brainstorm toolbox, applying default parameters (Gaser and Dahnke, 2016). For the quality control assurance checks, we relied on the control metrics generated by CAT12. Beyond that, the quality of the segmentation was visually checked. No MRI scans were excluded due to poor image quality. For the purpose of thalamic reconstruction we relied on an *in-vivo p*robabilistic atlas of human thalamic nuclei derived from diffusion-weighted MRI and implemented in CAT12 (Najdenovska et al., 2018). Thalamic segmentation and quantification of thalamic nuclei volumes was also repeated with Freesurfer (Dale et al., 1999), as a recent study shows it may have slightly better performances (Burggraaff et al., 2021). The Freesurfer procedure is based on a probabilistic atlas of the human thalamic nuclei achieved combining *ex vivo* MRI and histology (Iglesias et al., 2018). For both CAT12 and Freesurfer procedures, we also extracted the Total Intracranial Volume, to normalize (ratio) the volume of thalamic nuclei (Malone et al., 2015). The maps of cortical thickness were smoothed with a 5 mm Full Width at Half Maximum (FWHM) Gaussian kernel, thus choosing a rather conservative approach (Bernal-Rusiel et al., 2010; Brodoehl et al., 2020; Coalson et al., 2018; Hagler et al., 2006).

#### 2.4.2 MEG‐MRI co‐registration and source imaging

MEG data analysis was performed with the Brainstorm toolbox operating with MATLAB (Tadel et al., 2011). The cortical surfaces reconstructed via CAT12 were imported into Brainstorm software and downsampled to 15000 vertices (7500 per hemisphere). The reconstruction of the skull surfaces, as well as the co-registration of MEG-MRI data was performed using the original MRI in Brainstorm. The inverse solution procedure consists in computing a kernel to obtain single trial time-course reconstructions at the level of each vertex of the cortical surface. To do so, a subject-specific head model was built using the Boundary Element Method (BEM) available with the OpenMEEG toolbox (Gramfort et al., 2010). The conductivity value of the cortical surface was set to 0.33 S/m. For the solution of the inverse problem we applied the whitened and depth-weighted linear L2-minimum norm approach, assuming the orientation of the dipoles to be normal to the cortical mesh. Noise covariance for source reconstruction was calculated from a short resting state period (roughly 6 s) recorded prior to each MEG scan. Further methodological details on the procedures for MEG data acquisition and analysis can be found in our previous publications (Pellegrino et al., 2019a, 2019b; 2022; Pellegrino et al., 2018)

#### 2.4.3 MEG data preprocessing and ASSR data analysis

After third-order spatial gradient noise cancellation, data was downsampled to 600 Hz. Cardiac and eye movement artifacts were removed with the Signal Space Projection routine available in Brainstorm (Taulu and Simola, 2006; Tesche et al., 1995; Uusitalo and Ilmoniemi, 1997). SSP was preferred over ICA , since the latter may under some circumstances impair source reconstruction (Pellegrino et al., 2020). Note that as our interest was in 40 Hz auditory entrained gamma oscillations, data was filtered between 39 Hz and 41 Hz with a FIR filter prior to *ITPC* and *PLV* calculation. In order to minimize edge effects and avoid padding, data was split into epochs of 3 s, from -1.5 s to +1.5 s around the sound onset (Pellegrino et al., 2019a, 2019b). Each epoch was visually inspected and rejected if contaminated by artifacts. On average 3.46 (standard deviation = 2.82, min/max=0/9) trials per subject were removed after visual inspection.

We extracted measures of synchronization in a narrow frequency band between 39 and 41 Hz:

Inter-Trial Phase Consistency (*ITPC*): *ITPC* represents a local measure of phase consistency estimated across epochs. *ITPC* is bound between 0 and 1. The higher the *ITPC* is, the higher is the gamma synchronization at 40 Hz (Makeig et al., 2002). *ITPC* was estimated for the entire time-course (-1.5 to +1.5 s) and for each vertex of the cortical mesh. Then, *ITPC* values were converted into z-scores considering -500 to -200 ms as reference signal (Pfurtscheller and Da Silva, 1999). This reference/baseline time-window was selected to avoid proximity to the sound and edge effects. *ITPC* z-transformed measures were averaged in the 300-700 ms time-window, as after about 200 ms of 40 Hz sounds brain activity reaches a steady state of cortical synchronization increase (Larsen et al., 2018; Ross et al., 2003; Roß et al., 2000; Santarelli and Conti, 1999). This procedure resulted in one cortical map per recording.
The Phase Locking Value (*PLV*) is employed to calculate remote synchronization patterns between two distant brain regions. A higher value of *PLV* signifies higher connectivity, whereas a lower value means lower connectivity. A detailed mathematical description of *PLV* can be found in (Lachaux et al., 1999; Varela et al., 2001). *PLV*s were computed considering two seeds: left and right primary auditory cortices (A1). These two regions were extracted from the Desikan-Kiliany atlas (Desikan et al., 2006). In further details, *PLV* was computed between each vertex of the seed and each other vertex of the cortex. The maps of *PLV*s relative to vertices belonging to the seed were averaged. This was followed by z-transformation and time-averaging in a similar fashion to *ITPC*. Also in this case the output corresponded to a single map per seed indicating the degree (z-score) of *PLV* increase/decrease due to exposure to gamma sounds between the seed and the remaining cortical mantle. Since *PLV* has been shown to be sensitive to leakage and volume conduction (Colclough et al., 2016; Palva et al., 2018; Pellegrino et al., 2018;Vorwerk et al., 2014), we have applied another measure similar to *PLV* but less sensitive to these sources of bias – corrected imaginary Phase Locking Value (*ciPLV*) as suggested in Bruña et al. (2018).

Both *ITPC* and *PLV* maps were projected onto a template cortical surface extracted from the standard MRI (MNI ICBM152) (Mazziotta et al., 2001) and spatially smoothed with an FWHM Gaussian kernel of 5 mm. We additionally extracted raw *ITPC* values during stimulus exposure (auditory-driven) and Silence and computed the connectivity between the primary auditory cortices considering *PLV* and *ciPLV* (auditory-driven and Silence).

### 2.5 Statistical Analyses

Statistical analyses were performed with the Brainstorm and Fieldtrip toolboxes (Oostenveld et al., 2011). Cortical thickness was correlated with *ITCP* or *PLV* using Spearman's correlation coefficients, Fisher's z transformed, and corrected for multiple comparisons using a cluster based permutation approach (Monte-Carlo-Simulation) shuffling the data through 5000 permutations (Maris and Oostenveld, 2007). A similar approach was performed to investigate the relationship between the volumes of thalamic nuclei and gamma synchronization. The level of significance was set to *p* < 0.05. Considering the fact that 20 subjects were scanned twice in the course of the experiment, we ran the statistical analysis once with all scans (n=72) and once considering only one scan per subject (n=52). While the former results can be found in the main text, the latter results are depicted in Supplementary Fig. 1.

## 3 Results

### 3.1 Correlation between ITPC and cortical thickness

**Fig. 3:**
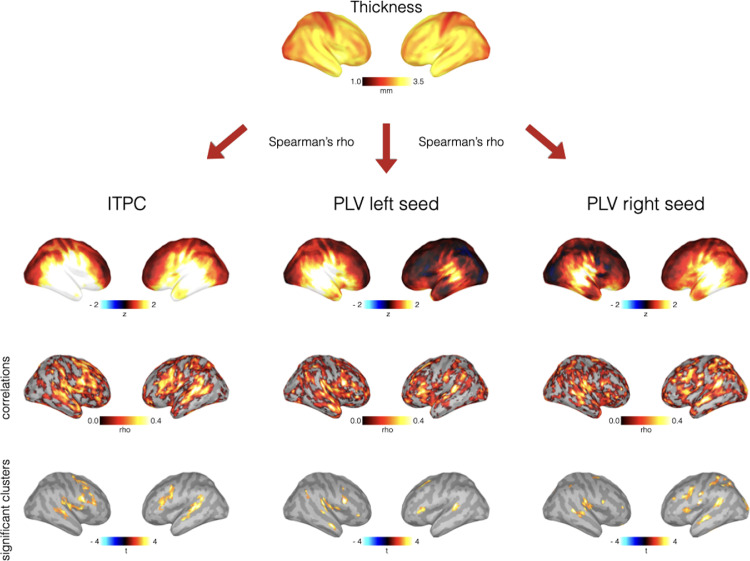
Overview of Main Findings. *Upper Panel. Cortical Thickness.* Cortical thickness is especially high in the temporal and prefrontal cortices. *Middle Panel. ITCP*, right seed *PLV* and left seed *PLV. ITPC* is high in the temporal and ventral frontal cortex. There were high positive *PLVs* for the (posterior) temporal lobes and negative *PLVs* in the frontal and occipital cortices on the sides of the respective seeds and high contralateral temporal lobe connectivity. *Lower Panels.* Correlations between *ITPC*/*PLVs* and cortical thickness. Rho was highest in frontal and temporal lobes for *ITPC* correlation. Significant clusters included regions of the left STG, left frontal cortex including left DLPFC and premotor cortex, right STG, right SMA and premotor cortex and DLPFC. Concerning *PLVs’* rho-values, they were high in the auditory cortices and frontal lobes. There was a significant positive correlation between cortical thickness and *PLV* with increased t-values in bilateral STG and frontal cortex for the respective seeds. For right A1 seed there were additionally increased t-values in contralateral parietal and occipital cortex.

Both cortical thickness and *ITCP* were higher in the temporal lobe and in the frontal lobe (Fig. 3). Induced *ITPC* raw scores were high in the temporal lobe, but there was no specific pattern during Silence (Supplementary Figure 4). Cluster-based permutation tests revealed a significant positive correlation between cortical thickness and *ITPC*, which corresponded to increased t-values in the left superior temporal gyrus (STG), left frontal cortex including left dorsolateral-prefrontal cortex (DLPFC) and premotor cortex, right superior temporal gyrus (STG), right supplementary motor area (SMA) and premotor cortex and DLPFC (Fig. 3).

By considering only one scan per subject (*n* = 52) significant clusters were found with increased t-values in the left superior temporal gyrus (STG) and right angular gyrus and SMA. Finally, we did not find any significant relationship between cortical thickness and *ITPC* during Silence.

### 3.2 Correlation between ITPC and volumes of thalamic nuclei

There was no significant correlation between thalamic nuclei volumes and cortical *ITPC*, neither for the CAT12 nor for the Freesurfer analysis (exemplary Freesurfer segmentation is provided in Supplementary Fig. 5).

### 3.3 Correlation between PLV and cortical thickness

*PLV* z-scores are depicted in Fig. 3. There were high positive *PLV*s for the (posterior) temporal lobes and negative *PLV*s in the frontal and occipital cortices on the ipsilateral sides of the respective seeds. Concerning the contralateral hemispheres, *PLVs* were high in the temporal, parietal and frontal lobes. Cluster-based permutation tests revealed a significant positive correlation between cortical thickness and *PLV* with increased t-values in STG and frontal cortex for the respective auditory seed in the ipsilateral hemispheres. Concerning contralateral *PLVs*, cluster-based permutation tests revealed a significant positive correlation between cortical thickness and left A1 seed in the right perisylvian areas. A similar pattern could be found for the right A1 seed with additional increased t-values in the left dorsal prefrontal cortex, parietal and occipital lobes. The rho-values where high in frontal cortex, and temporal lobe for both seeds. Concerning the sub-sample of n=52, there were increased t-values in bilateral frontal and temporal lobe for left seed and increased t-values in the right temporal and frontal lobe and left temporal, frontal, occipital and parietal lobe for right seed.

Results for *ciPLV* are depicted in Supplementary Figs. 2 & 3. Left and right A1 seed *ciPLVs* resulted in significant correlations with cortical thickness, featuring increased t-values in bilateral frontal, temporal, parietal and occipital lobes.

**Fig. 4:**
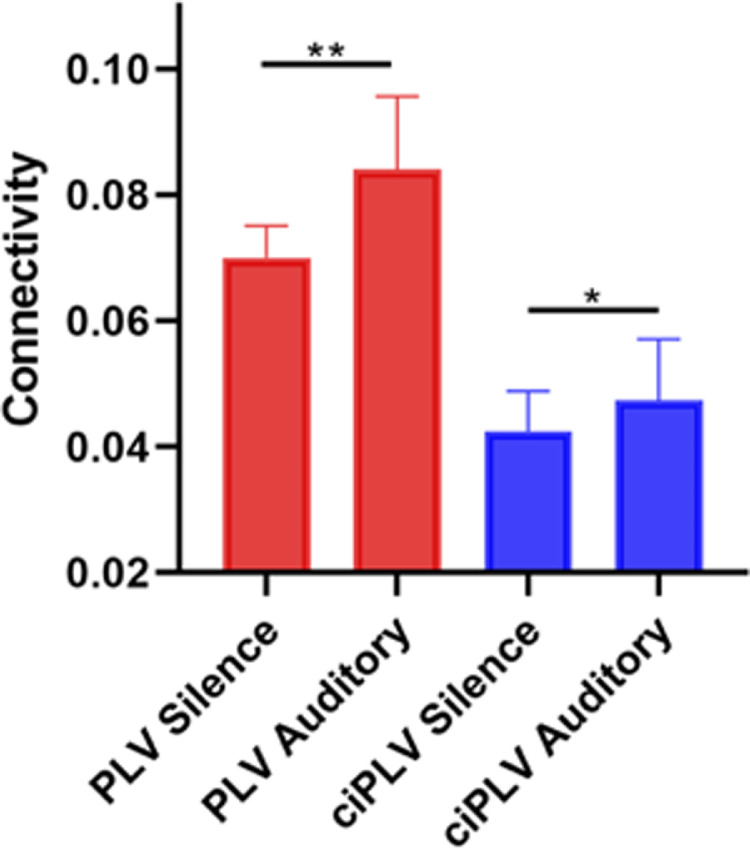
Connectivity between left and right A1 ROI. *PLV* and *ciPLV* were significantly increased during auditory stimulation as compared to Silence. Displayed are median and interquartile ranges.

Connectivity between primary auditory cortices increased during auditory exposure as compared to Silence for both *PLV* and *ciPLV* (*PLV* Z = 5.539, *p* < 0.001; *ciPLV* Z = 2.457, *p* = 0.013; Fig. 4).

## 4 Discussion

In this study, a significant correlation between gamma synchrony and cortical thickness has been revealed. The relationship was specific for auditory-driven gamma synchrony and was not found for gamma synchrony at rest (during Silence). The correlation was not limited to the primary auditory cortex (A1) (Edgar et al., 2014; Hirano et al., 2020; Kim et al., 2019), but rather involved widespread cortical areas and, especially, the frontal cortex in the premotor area, SMA and DLPFC. The relationship between synchrony and thickness was twofold: thickness had in fact an impact on local gamma synchrony (*ITPC* measure), and also influenced gamma connectivity across parts of the cortical surface (i.e. *PLV* between auditory cortices taken as seeds and remaining cortical surface areas of both hemispheres).

A relationship between thickness and gamma synchrony has already been investigated in restricted cortical regions and, despite some conflicting results, it was found in the temporal cortex in relation to auditory gamma stimulation (Edgar et al., 2014; Hirano et al., 2020; Kim et al., 2019), in the occipital cortex in relation to visual stimulation (Gaetz et al., 2012; Muthukumaraswamy et al., 2010; van Pelt et al., 2018) and in the somatosensory cortex following electrical stimulation of peripheral nerves (Proskovec et al., 2020). We took advantage of auditory entrainment of gamma synchronization over parts of the cortical surface in the temporal and frontal lobes to replicate and expand previous evidence that variations in cortical cell density are involved in the generation of gamma synchrony in widespread cortical regions (Fig. 3) (Farahani et al., 2021; Koshiyama et al., 2020; Pellegrino et al., 2019b; Tada et al., 2021).

The topographical distribution of the structure-function relationship showed a remarkable specificity, with the strongest effects in the temporal and frontal lobes. Here, the correlation coefficients reached values as high as rho=0.5 for both *ITPC* and *PLV* (Figure 3). This specific pattern may be due to cytoarchitectonic peculiarities and the strong connections between frontal regions and temporal cortex. In detail, one fourth of GABAergic neurons of the primate DLPFC are parvalbumin positive (Condé et al., 1994; Gabbott and Bacon, 1996) and experimental studies support an association between gamma synchrony and parvalbumin interneurons in this region (Cho et al., 2015, 2020). The ventral part of the premotor cortex is functionally connected with the primary auditory cortex (Genon et al., 2018) and the interaction between ventral premotor cortex and auditory cortex is involved in rhythm perception (Chen et al., 2009). The connection between centro-frontal areas and temporal cortex has been demonstrated at multiple levels: stimulation of the central regions results in consistent effects of gamma inhibition in the temporal lobe (Chen et al., 2009; Pellegrino et al., 2019b).

Finally, the level of gamma entrainment has been associated with executive functioning in health and disease, in humans and animals (Cho et al., 2015, 2020; Fries, 2009; Parciauskaite et al., 2021). Taken together a significant and strong structure-function relationship over widespread cortical regions for auditory gamma entrainment could be demonstrated here. Interestingly, the relationship between gamma synchrony and thickness was specific to auditory-driven activity and was not found at rest (during Silence). This result replicates the findings of our previous studies (Larsen et al., 2018; Pellegrino et al., 2019b) and is also in agreement with the finding that in most neuropsychiatric conditions with gamma impairment the level of gamma synchronization at rest is not affected. Another approach suggests that the interneuron system underlying gamma synchrony is responsible for down-regulation of neural activity during rest as well as up-regulation as a consequence of stimulus processing (Gandal et al., 2012; Sohal et al., 2009) underlining the fact that gamma synchrony is triggered by external stimuli rather than rest.

Moreover, in a recent study (Mahjoory et al., 2020), investigating the posterior-anterior (visual) gradient in resting state MEG and its relationship with cortical thickness gradients an opposite pattern was found for functional (alpha band) vs. structural (cortical thickness) gradients. The authors did not report gamma oscillations, since they were absent in most cortical areas in their analysis, and conclude that a different data processing approach would be required in order to capture gamma gradients. In another study investigating the occipital cortex (van Pelt et al., 2018), it was reported that peak gamma frequency due to visual stimulation was positively correlated with cortical thickness. Concerning gradient patterns for resting state as well as gamma frequency stimulation, this relationship remains to be established in auditory and visual areas.

There was no correlation between volumes of thalamic nuclei and *ITPC*. A role of subcortical nuclei in the generation of synchronous gamma activity is certain (Chaitanya et al., 2020). For instance, patients with small subcortical stroke lesions show a widespread impairment of generation of gamma activity in phase (Pellegrino et al., 2019a). Nonetheless, in our cohort of healthy subjects we did not find a relationship between the volume of thalamic nuclei and gamma synchrony, after correction for multiple comparisons. We had simplistically hypothesized that the bigger the thalamic nuclei, the better would have been cortical synchrony. While this hypothesis is reasonable and was verified for cortical thickness, it did not take into account the complex relationship between thalamic activation and cortical function. Further work on the relationship between thalamic volume and cortical gamma synchrony is nonetheless needed.

### 4.1 Connectivity and gamma synchrony

Distant brain areas show some degree of coherent gamma activity, potentially explaining our significant results on *PLV* (Adaikkan and Tsai, 2020; Bosman et al., 2012; Fries, 2009; Gregoriou et al., 2009; Igarashi et al., 2014; Rohenkohl et al., 2018; Yamamoto et al., 2014). Our data demonstrates that cortical thickness not only has an impact on the generation of gamma synchrony, but also on its propagation throughout parts of the cortical surface. This relationship has been found by calculating *PLV*, which measures the degree of phase alignment across brain regions and is a connectivity measure. *PLV* was computed for each A1 against the rest of the cortical surface. There was a positive correlation between thickness and *PLV* in left inferior frontal gyrus for left and right A1, possibly reflecting structure-function relationships between linguistically relevant areas (Hertrich et al., 2020). Moreover, there were positive correlations between the seeds and areas in the frontal and parietal lobes and left occipital lobe for the right seed, implying a functional relationship between these areas that is strongly associated with structure (potentially the content of interneurons).

### 4.2 Spatial and temporal coherence of gamma synchrony

Here we could show that an association between gamma synchrony and cortical thickness beyond A1 does not only emerge in the temporal domain (*ITPC*) but is also reflected in spatial relation (*PLV*). Using Granger causality analysis, Koshiyama et al. (2020) revealed effective ASSR-related connectivity in widespread cortical areas underlining the functional dependency between these areas. The association between gamma synchrony and cortical thickness, which reflects structure-function relationship between temporal and other lobes, might be mediated by structural connectivity of these areas e.g. by the fasciculus arcuatus (De Vos et al., 2020; Vandermosten et al., 2013).

### 4.3 Comparison between *n* = 72 and *n* = 52

Some of our subjects were measured twice. To account for a potential bias introduced by considering all scans (n=72) as independent, we have repeated our analyses for unique subjects (n=52). Our results turned out to be robust to a lower sample size.

### 4.4 Volume conduction and inverse solution leakage

The effects of volume conduction and inverse solution leakage have been of concern in basic research comparing source reconstruction in MEG vs. EEG and clinical applications when it comes to presurgical planning (Aydin et al., 2020; Hedrich et al., 2017). The literature seems to agree that the only reliable way to mitigate this issue for phase-based connectivity measures is to exclude lag=0 connectivity (Colclough et al., 2016; Palva et al., 2018), however with the risk of missing real lag=0 connectivity (Nolte et al., 2004). Therefore, we opted here to apply both *PLV* and *ciPLV* metrics to our data and compare them.

Interestingly, by applying the two different measures we demonstrated that the connectivity between primary auditory cortices and whole cortical mantle occurred simultaneously (*PLV*) and with some degree of delay (*ciPLV*). Cortical thickness was significantly and positively correlated with both, suggesting a role for local generation of synchronous activity as well as for multiple ways of oscillatory propagations. The spatial distribution of correlation based on *PLV* and *ciPLV* is different in pattern.

Moreover, functional connectivity between the primary auditory regions increases during exposure to 40 Hz auditory modulated tones as compared to Silence. Both *PLV* and *ciPLV* exhibited a similar behavior suggesting that the primary cortices interact simultaneously (*PLV*, lag=0) and with some degree of delay (*ciPLV*, lag≠0).

### 4.5 Gamma entrainment – behavior and translational value

While the general message of this study is the relevance of structural properties in the generation of gamma activity in phase, it should not be neglected that simple functional manipulation of cortical excitability and efficiency of GABA transmission has an effect on gamma synchrony as well, even at distance from the stimulated region. For instance, our group has demonstrated that tDCS of the sensory-motor cortex attenuates gamma synchrony in the perisylvian areas of the cathode side (right in that case) (Pellegrino et al., 2019b). This is justified by the effect of non-invasive brain stimulation on GABAergic and glutamatergic neuro-transmission (Antal et al., 2010; Clark et al., 2011; Hummel et al., 2005; Kidgell et al., 2013; Nitsche et al., 2005; Stagg et al., 2009; Zhang et al., 2014). Interestingly, while there have been multiple attempts to intervene on gamma generation so to obtain a behavioral gain, recent evidence also suggests that gamma sensory stimulation itself may have a therapeutic effect. Gamma induction may be neuroprotective against neurodegenerative diseases (Adaikkan et al., 2019; Martorell et al., 2019) and animal models of Alzheimer's disease have allowed to demonstrate that repetitive sensory gamma entrainment improves the function of fast-spiking parvalbumin-positive interneurons as well as microglia. A study involving human Alzheimer's patients has recently shown that regular treatment with sensory 40 Hz gamma attenuated ventricular dilation and hippocampal atrophy, stressing the role of gamma entrainment on brain structure (Chan et al., 2021). In other words, if our study supports the idea that structure impacts on function (gamma synchrony), other groups have demonstrated that this link may also work the other way around, and that repetitive functional gamma stimulation can preserve cortical structure especially in the field of neurodegeneration. Other models of neuropsychiatric conditions are of further support of this perspective. Indeed, aberrant gamma synchrony has been shown repeatedly in schizophrenia (Brenner et al., 2009; Koshiyama et al., 2021; Kwon et al., 1999; Light et al., 2006; Puvvada et al., 2018; Vierling-Claassen et al., 2008), which has mainly been attributed to inefficient GABAergic neurotransmission in the DLPFC (Sun et al., 2011). In fact, the number of interneurons has been shown to be decreased in some schizophrenic patients (Hashimoto et al., 2003; Lewis et al., 2005). Investigating the association between gamma synchrony and cortical thickness might allow for conclusions on the role of underlying cell architecture.

### 4.6 Limitations and outlook

As discussed above we have performed analyses including two scans of some subjects, however assuming them to be independent. We have accounted for this by recalculating the same analysis only including one scan per subject, and overall results were comparable.

Moreover, we have used a connectivity measure (*PLV*) which is prone to correlations due to volume conduction and source leakage. To account for this, we have applied another measure (*ciPLV*) which neglects zero-lag connectivity, but is insensitive to the mentioned sources of bias. There is a certain trade-off neglecting zero-lag connectivity considering the temporal resolution of the assumed true connectivity. However, by comparing the two measures, we were able to find a compromise between the advantages and disadvantages of the two metrics.

In future studies, the relationship between cortical thickness and gamma synchrony in patient populations remains to be revealed. In this respect, gamma synchrony has been associated with rehabilitation outcome in stroke patients (Pellegrino et al., 2019a) and cortical thinning has been found in remote areas to primary stroke lesions (Cortese et al., 2021). The association between cortical thinning and gamma synchrony might therefore provide valuable information about network effects of structural and functional recovery.

## 5 Conclusions

In this study, we have investigated the association between gamma synchrony and cortical thickness as well as the correlation between connectivity and cortical thickness. Both functional values were linked to the brain structure in widespread cortical areas, especially in the frontal lobe, indicating an involvement of neural cells beyond A1 in the generation of gamma synchrony. Gamma entrainment and cortical thickness may favor each other explaining e.g. neuroprotective effects of gamma entrainment therapy.

## CRediT authorship contribution statement

**Anna-Lisa Schuler:** Methodology, Formal analysis, Visualization, Writing – original draft, Writing – review & editing, Validation. **Giulio Ferrazzi:** Methodology, Formal analysis, Writing – review & editing, Validation. **Nigel Colenbier:** Methodology, Formal analysis, Writing – review & editing, Validation. **Giorgio Arcara:** Investigation, Data curation, Writing – review & editing. **Francesco Piccione:** Resources, Funding acquisition, Project administration, Writing – review & editing. **Florinda Ferreri:** Writing – review & editing, Validation. **Daniele Marinazzo:** Methodology, Formal analysis, Writing – review & editing, Validation, Funding acquisition. **Giovanni Pellegrino:** Conceptualization, Methodology, Formal analysis, Visualization, Writing – review & editing, Funding acquisition, Supervision.

## Declaration of Competing Interest

The authors have no conflict of interest to disclose.

## Appendix Supplementary materials

Image, application 1
