# Supplementary material for: Auditory driven gamma synchrony is associated with cortical thickness in widespread cortical areas
Source: Neuroimage. 2022 Jul 15;255:119175. doi: 10.1016/j.neuroimage.2022.119175 (PMC9168448; doi:10.1016/j.neuroimage.2022.119175)
Supplement: Supplementary file 1 [file mmc1.docx]

**Supplementary Material**

We repeated the analysis of ITPC, PLV and their correlation with cortical thickness for 52 subjects, excluding those that were scanned twice. The parameters are the same as those reported in the main text.


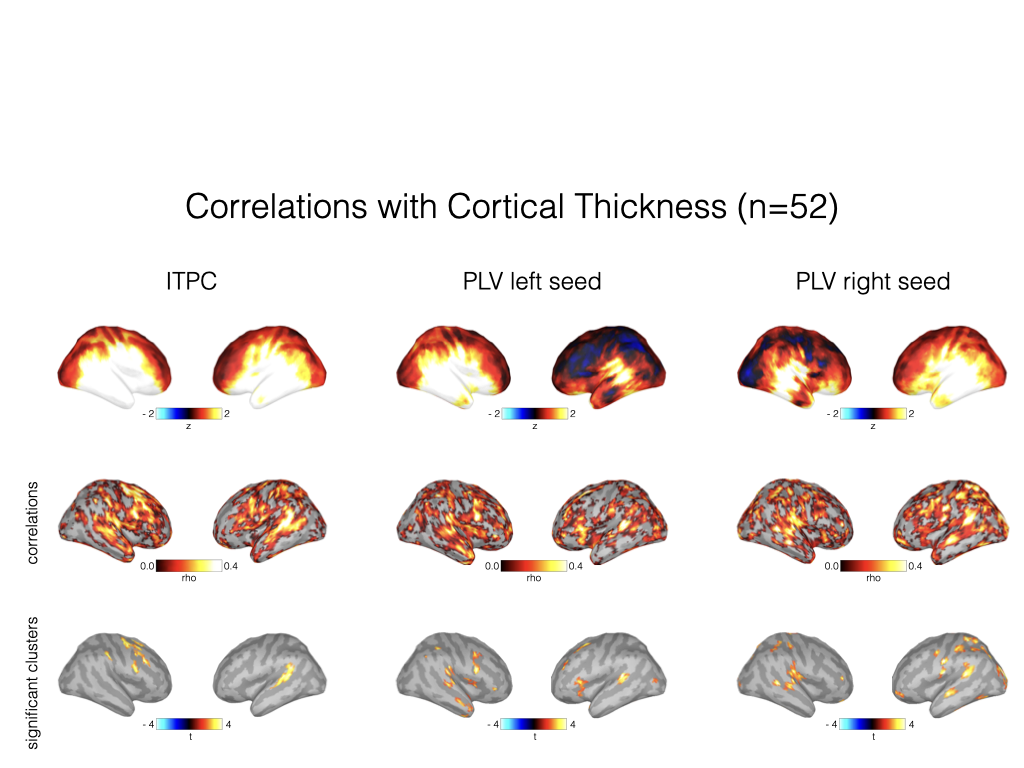


Supplementary Figure 1. **ITPC cortical thickness correlations for n=52.** From left to right, ITPC, PLV from left A1 and PLV from right A1. From top to bottom: average z-scores (auditory exposure vs. Silence) across 52 subjects, map of rho-values and clusters of significant structural-functional correlation. There was a significant correlation between ITCP/PLV and cortical thickness. For ITPC significant clusters were found with increased t-values in the left superior temporal gyrus (STG) and angular gyrus and SMA. For left A1 seed there were increased t-values in bilateral frontal and temporal lobe. Concerning right A1 seed there were increased t-values in the right temporal and frontal lobe and left temporal, frontal, occipital and parietal lobe.

In order to account for leakage and volume conduction (Colclough et al., 2016; Palva et al., 2018; Vorwerk et al., 2014), we have applied a version of PLV which is less sensitive to these sources of bias – corrected imaginary Phase Locking Value (ciPLV) as suggested in Bruña et al. (2018). In the course of this study we have implemented the ciPLV into the Brainstorm toolbox^[[1]](#footnote-1)^.


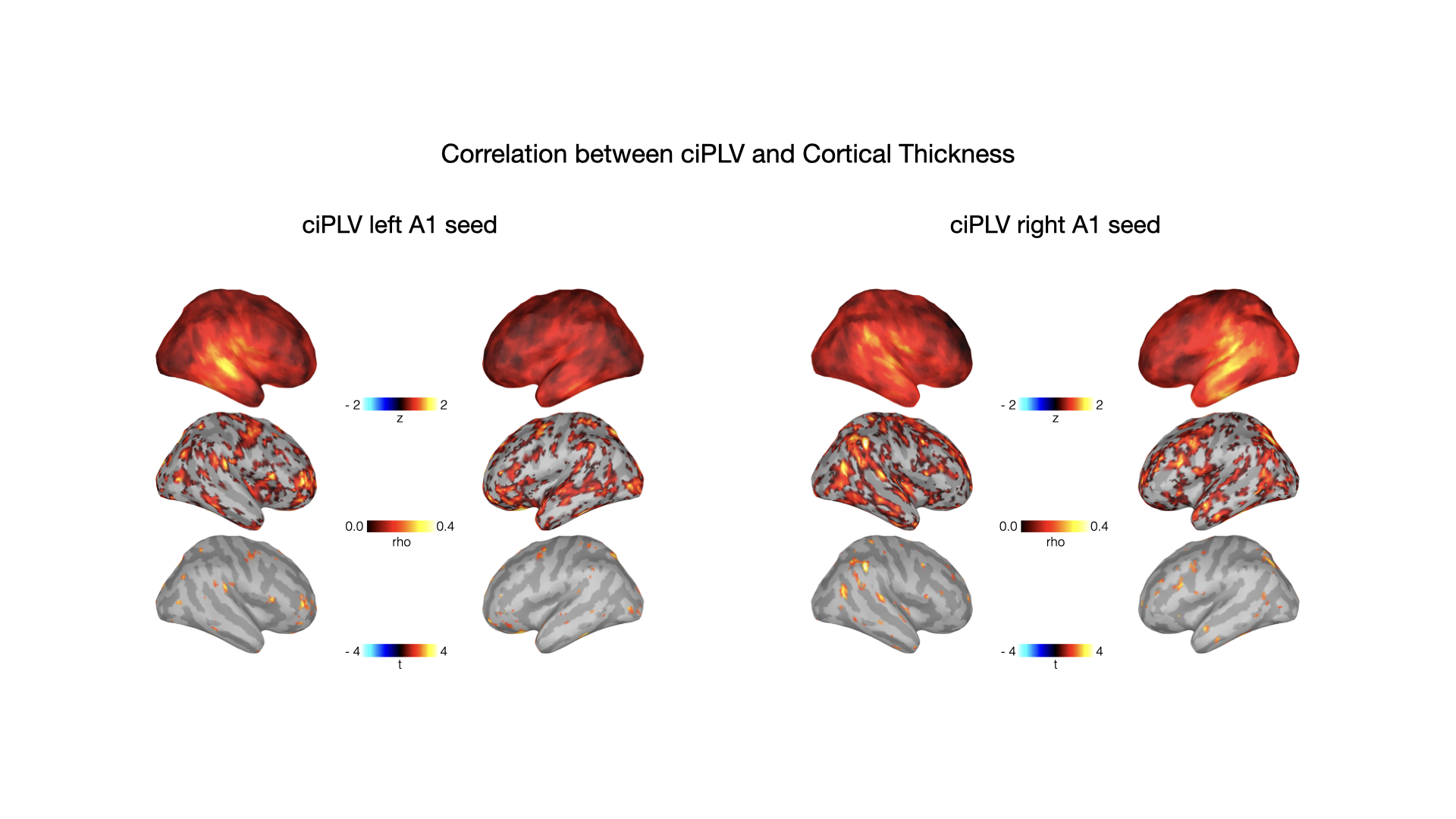


Supplementary Figure 2**. ciPLV and cortical thickness.** Left and right A1 seed ciPLVs resulted in significant correlations with cortical thickness, featuring increased t-values in bilateral frontal, temporal, parietal and occipital lobes.

*Supplementary Figure 3****. Comparison between ciPLV/PLV correlations with cortical thickness.*** There was a positive correlation between cortical thickness and connectivity for ciPLV and PLV. The comparison between the two metrics revealed one instantaneous (lag=0) correlation which was captured with PLV (and which is also reflected by the behavior of ITPC) and one non-instantaneous (lag≠0) correlation which was captured with ciPLV.


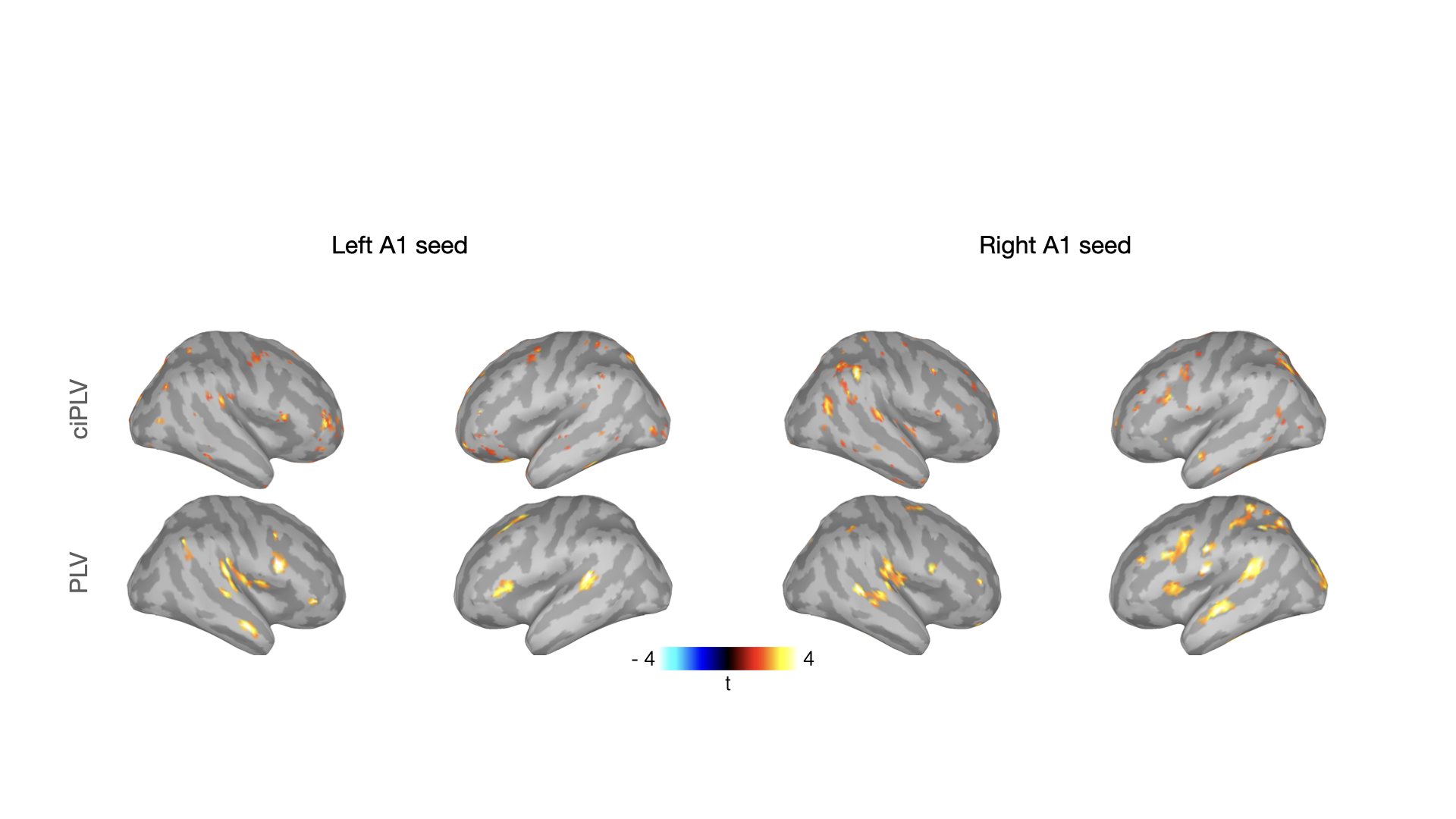


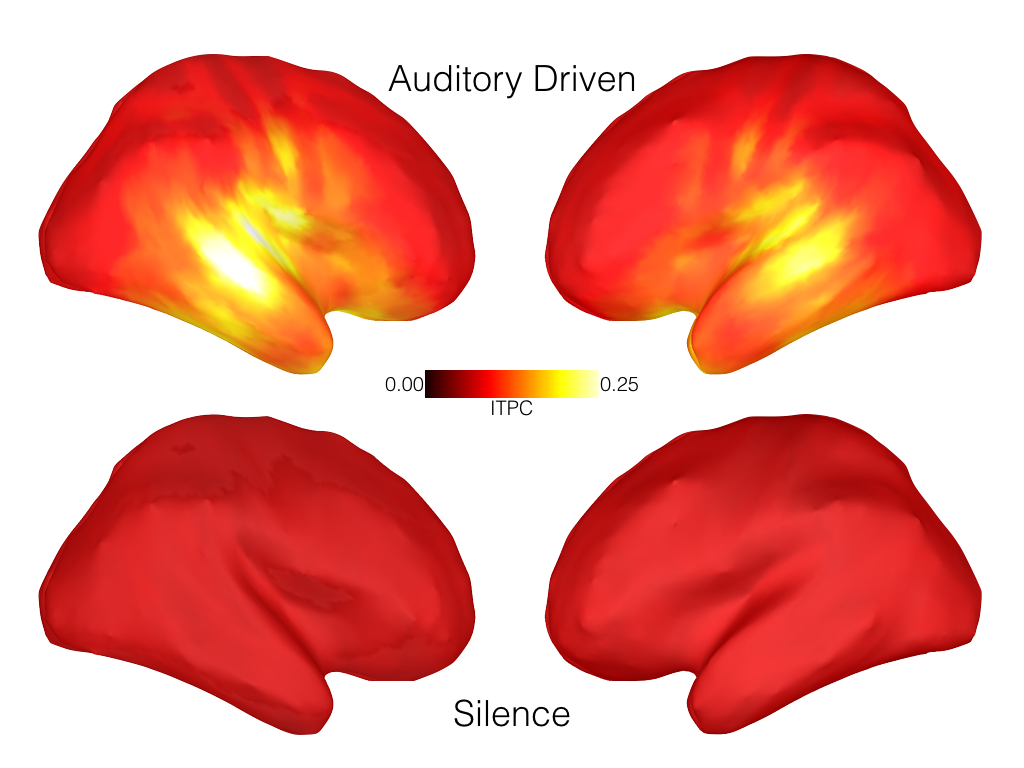


Supplementary Figure 4**. Comparison between induced ITCP and ITPC at Silence.** While there is increased 40 Hz ITPC in the primary auditory region, no such effect is seen during Silence.


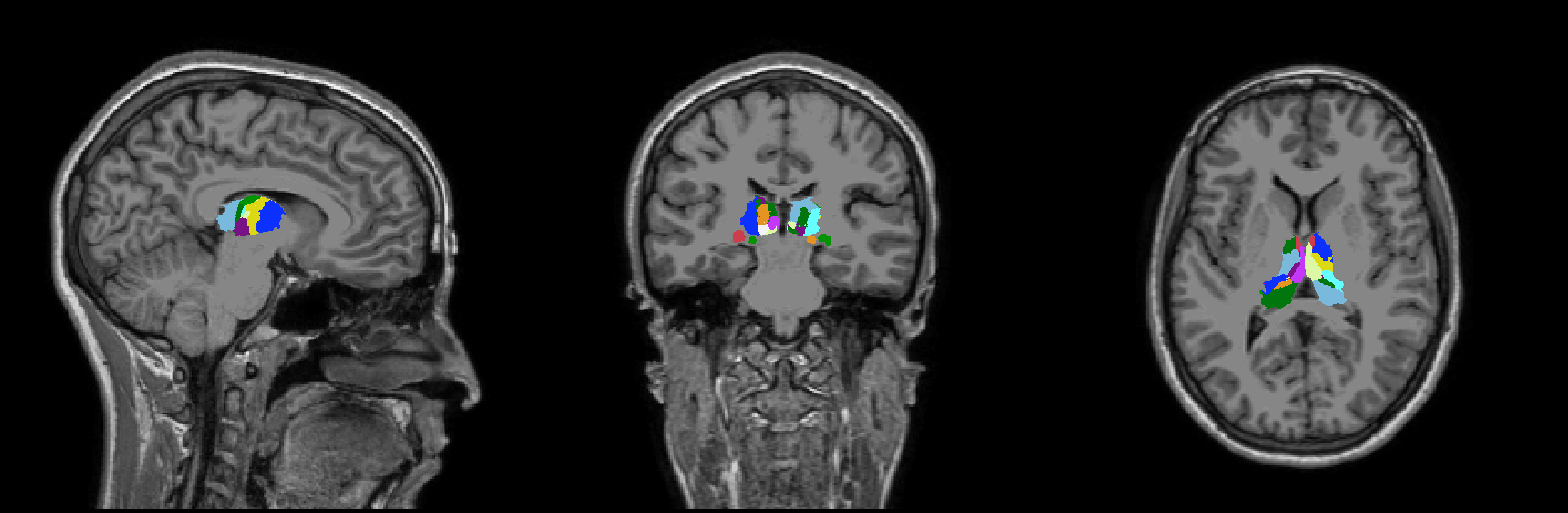


Supplementary Figure 5. **Thalamic Nuclei.** Exemplary subject for the segmentation of thalamic nuclei using Freesurfer.

References

Bruña, R., Maestú, F., Pereda, E., 2018. Phase locking value revisited: teaching new tricks to an old dog. Journal of neural engineering 15, 056011.

Colclough, G.L., Woolrich, M.W., Tewarie, P., Brookes, M.J., Quinn, A.J., Smith, S.M., 2016. How reliable are MEG resting-state connectivity metrics? Neuroimage 138, 284–293.

Palva, J.M., Wang, S.H., Palva, S., Zhigalov, A., Monto, S., Brookes, M.J., Schoffelen, J.-M., Jerbi, K., 2018. Ghost interactions in MEG/EEG source space: A note of caution on inter-areal coupling measures. Neuroimage 173, 632–643.

Vorwerk, J., Cho, J.-H., Rampp, S., Hamer, H., Knösche, T.R., Wolters, C.H., 2014. A guideline for head volume conductor modeling in EEG and MEG. NeuroImage 100, 590–607.

1. https://github.com/brainstorm-tools/brainstorm3/commit/43904a5e299cfae5c2dfe70bee48aa6691b92aea [↑](#footnote-ref-1)
